# The expression and methylation status of vitamin D receptor gene in Behcet's disease

**DOI:** 10.1002/iid3.275

**Published:** 2019-11-10

**Authors:** Sam Seydi Shirvani, Mohammad Nouri, Ebrahim Sakhinia, Zohreh Babaloo, Golamreza jadideslam, Alipour Shahriar, Jafar Farhadi, Alireza khabbazi

**Affiliations:** ^1^ Molecular Medicine Department, Connective Tissue Diseases Research Center Tabriz University of Medical Sciences Tabriz Iran; ^2^ Biochemistry Department Tabriz University of Medical Sciences Tabriz Iran; ^3^ Genetic Department, Connective Tissue Diseases Research Center Tabriz University of Medical Sciences Tabriz Iran; ^4^ Immunology Department, Connective Tissue Diseases Research Center Tabriz University of Medical Sciences Tabriz Iran; ^5^ Molecular Medicine Department, Connective Tissue Diseases Research Center Tabriz University of Medical Sciences Tabriz Iran; ^6^ Biochemistry Department Urmia University of Medical Sciences Urmia Iran; ^7^ Molecular medicine Department Tabriz University of Medical Sciences Tabriz Iran; ^8^ Connective Tissue Diseases Research Center Tabriz University of Medical Sciences Tabriz Iran

**Keywords:** Behcet's disease, epigenetic, gene expression, methylation, vitamin D receptor (VDR)

## Abstract

**Introduction:**

Vitamin D has important roles as a natural immune modulator via regulating the expression of genes which have been implicated in the pathophysiology of autoimmune diseases. Vitamin D function and its deficiency have been linked to a wide range of metabolic disorders including disorders of calcium metabolism, malignant, cardiovascular, infectious, neuromuscular, and inflammatory diseases. Environmental factors, genetic factors, and epigenetic changes contribute to Behcet's disease (BD) development. The aim of our study was to analyze the expression level and methylation status of the vitamin D receptor (VDR) gene promoter in the peripheral blood mononuclear cells (PBMCs) of patients with BD.

**Methods:**

In a case‐control study, 48 Iranian Azeri patients with BD and 60 age‐, sex‐ and ethnically‐matched healthy controls were included. Venous blood samples were collected and PBMCs were isolated by Ficoll protocol. The DNA and RNA were subsequently extracted. Promoter methylation levels were evaluated by MeDIP‐quantitative polymerase chain reaction (qPCR). The expression of VDR was evaluated by real‐time PCR.

**Results:**

The results of quantitative real‐time PCR analysis showed that the level of VDR expression in patients with BD was significantly lower than the control group (*P* = .013). There was no significant difference in the level of DNA methylation in the BD and control groups (*P* > .05). As the results show, the expression level of *VDR* gene was significantly different between female and male in the patient group (*P* = .001). *VDR* gene expression was significantly higher in subjects with phlebitis. No correlation was observed between *VDR* gene expression rate and BD activity.

**Conclusion:**

*VDR* gene expression decreased in patients with BD. However, there is no suggestion evidence that the expression level of VDR is regulated by a unique DNA methylation mechanism. No correlation exists between *VDR* gene expression and BD activity.

## 1 INTRODUCTION

Behcet's disease (BD) is a chronic relapsing multisystem inflammatory disease characterized by recurrent oral aphthous ulcers, genital ulcers, and uveitis. Variable skin lesions, inflammatory arthritis, vasculitis, epididymitis, and central nervous system and gastrointestinal tract involvement are other symptoms. BD often occurs in young patients aged between 20 and 40 years.^1^ The etiology of BD is unknown. However, environmental factors especially microbial agents,^2^, ^3^ vitamin D deficiency,^4^ and possibly smoking,^5^ genetic factors,^6^, ^7^, ^8^, ^9^, ^10^, ^11^ and epigenetic changes^12^ contribute to disease development.

Vitamin D is a fat‐soluble vitamin that is essential for health. Less than 10% of vitamin D is ingested through food; the rest is produced in the deep epidermis layer of the skin under the effect of sunlight.^13^ The genes involved in the regulation and synthesis of vitamin D include 25‐hydroxylase (*CYP2R1*), 1α‐hydroxylase (*CYP27B1*), 24‐hydroxylase (*CYP24A1*), and *DHCR7*.^13^, ^14^, ^15^ Vitamin D exists in two major physiologically forms: 25‐hydroxyvitamin D3 (25[OH] D3) that is obtained from UV irradiation and 1α, 25 dihydroxyvitamin D3 (1α, 25[OH] 2D3) that is synthesized in the skin. In the skin, this form of Vitamin D is synthesized from 7‐dehydrocholesterol when exposed to UVB.^16^

Of these two types, 25 (OH) D3, which represents the main circulating vitamin D metabolite and is the most reliable parameter to define human vitamin D status. However, 25(OH)D3 requires a further hydroxylation in the kidneys by the 1α‐hydroxylase (CYP27B1) to form the biologically active form of vitamin D 1α, 25(OH) 2D3. The other form of vitamin D; that is, 1α, 25(OH) 2D3—the biologically active form of vitamin D—acts as a pleiotropic endocrine hormone.^13^

Vitamin D acts through binding to vitamin D receptor (VDR). VDR is one of the DNA‐binding transcription factors of the nuclear receptor superfamily, which mediate biologically active forms of vitamin D (1α, 25(OH)2D3) signaling.^17^ 1α, 25(OH)2D3 after binding to the VDR complex in target cells, forms heterodimers with retinoid x receptor (RXR), translocate to the nucleus and binds to vitamin D response elements (VDREs), modulates the transcription of target genes and leads to specific biological response.^16^, ^18^ These responses are tissue‐specific and include control of the bone metabolism, growth, differentiation, and functional activity of numerous cell types including those of the immune system, skin, the pancreas, and bone. Binding of 1,25(OH)2D3 to the intracellular VDR regulates more than 900 genes.^19^ The ubiquitously expression of *VDR* gene by almost all tissues and cells of body provides a biological basis for widespread functions of VDR and its ligands.^20^ The VDR encoding gene is located on the long arm of chromosome12 (12q13.11), contain eight introns, nine exons with four potential promoter regions (Exons1a, 1c, and 1d of the VDR are well conserved, while1b, 1e, and 1f show low homology).^14^ Exon1a has a strong promoter, counting several transcription factor‐binding sites (AP‐2 and SP1). *VDR* gene polymorphisms have been implicated in mediating susceptibility to many autoimmune and autoinflammatory diseases.^11^, ^19^, ^21^, ^22^, ^23^ The most studied VDR polymorphisms are TaqI, BsmI, ApaI, and FokI.^14^, ^16^ Kolahi et al.^11^ showed the association between FokI polymorphism of VDR and BD in the Azari population of Iran. Tizaoui et al.^24^ reported the association between TaqI and ApaI polymorphism of the *VDR* gene and BD in Tunisians. DNA methylation is one of the epigenetic mechanisms that have an important role in regulation of genes expression.^14^ In humans, DNA methylation occurs on CpG islands (CGI) of promoter regions.^25^ Methylation of DNA is mediated by DNA methyltransferases (DNMTs) enzymes^26^ and usually linked with gene silencing.^23^ Recent studies have shown that the methylation status of CpG islands of *SOCS1* and *IL‐10* genes is related to the pathogenesis of BD.^27^, ^28^ Several studies have suggested that *VDR* gene methylation alterations may have a role in the pathogenesis of infectious and malignant diseases.^29^, ^30^, ^31^, ^32^, ^33^, ^34^

Vitamin D plays an important regulatory role in immune system^6^ function and its deficiency has been introduced in the etiology of autoimmune diseases.^19^, ^35^ It affects many aspects of the innate and adaptive immune system.^36^ Vitamin D deficiency causes enormous changes in innate immune system: (a) macrophages maturation impairment, (b) increase in the production of IL12 and decrease in the production of IL10, and (c) induction of cathelicidins of monocytes and macrophages.^19^ Vitamin D effects on adaptive immunity are: (a) prevention of monocyte‐macrophage lineage differentiation into dendritic cells and decreasing antigen presentation, (b) skewing the Th1/Th2 balance to Th2 response and suppressing Th17 response by enhancing the production of interleukin‐4 (IL‐4), IL‐5, and IL‐10, (c) increasing the regulatory T lymphocyte compartment, (d), inhibiting pro‐inflammatory cytokines production such as IL‐2, INF‐γ, IL‐17, and IL‐21, and (e) inhibiting B cells proliferation, plasma cell differentiation, and immunoglobulin secretion.^36^ The findings from Mediterranean countries, the Middle East, and the Far East indicate an inverse relationship between vitamin D and BD with higher levels of vitamin D correlating with lower levels of disease activity.^13^

The vast majority of studies about vitamin D and BD have focused on vitamin D status and polymorphisms of VDR. Very limited data exist about the epigenetic regulation of genes in BD. The aim of our study is to analyze the expression level and methylation of CpG sites in the *VDR* gene promoter of peripheral blood mononuclear cells (PBMC) in patients with BD.

## 2 MATERIALS AND METHODS

### 2.1 Study design and sample size

The study design was approved by the Ethics Committee of Tabriz University of Medical Sciences (TUMS). All subjects gave written informed consents before joining the study. Then patients were recruited consecutively from the BD clinic of Connective Tissue Diseases Research Center (CTDRC) between March 2016 and November 2017.

Patients group comprised of cases who were referred to Behcet's clinic and diagnosed with Behcet's disease according to international study group criteria (ISG) for BD. Patients’ characteristics including the main demographic features, disease duration, and clinical manifestations were registered through patients’ files and Behcet's clinic registry. BD activity was measured by Iranian Behcet's Disease Dynamic Activity Measure (IBDDAM)^37^, ^38^ and Behcet's Disease Current Activity Form (BDCAF).^39^

We determined our sample size based on the study of JE Do et al.^40^ To calculated sample size, we considered significance level of *P* < .05, power of 80% and 10% dropout. Totally, 60 participants in two groups included in the study.

**Figure 1:**
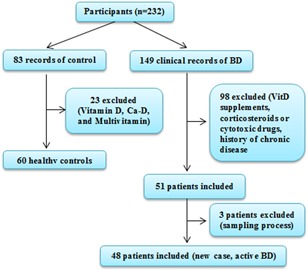
Study enrollment flowchart. We evaluated 149 consecutive clinical records of BD for inclusion and exclusion criteria. Exclusion criteria for BD subgroup consisted of vitamin D supplements, corticosteroids or cytotoxic drugs in the past 6 months. Moreover, patients with history of chronic disease were excluded from study. Exclusion criteria for control group consisted of vitamin D, Ca‐D, and multivitamin in the last 6 months. Finally, 48 eligible records with active BD and 60 ages‐, sex‐, and ethnically‐matched healthy controls were included. BD, Behcet's disease

In a case‐control study, we retrospectively evaluated 149 consecutive clinical records of BD patients for inclusion and exclusion criteria. Exclusion criteria for BD subgroup consisted of history of taking any kind of vitamin D supplements, any kind of corticosteroids or cytotoxic drugs in the past 6 months. Moreover, patients with history of chronic disease including renal or hepatic diseases, bone metabolic disease, malabsorption, type 1 diabetes mellitus, pregnancy, thyroid, and parathyroid disorders, malignancies were excluded from study. Furthermore, controls group with history of vitamin D, Ca‐D, and multivitamin in the last 6 months were excluded. Finally, 48 eligible records with active BD and 60 ages‐, sex‐ and ethnically‐matched healthy controls were included (Figure 1).

### 2.2 Sampling

The blood samples of the BD group were obtained between March 2016 and November 2017 and the blood samples of the control group were obtained in November 2017. PBMC was isolated by Ficoll‐Hypaque (Lymphodex, Inno‐Train, Germany) density gradient centrifugation and plasma was obtained after centrifugation and stored at −20°C until use.

#### 2.2.1 DNA, RNA isolation, and RT‐PCR method

Genomic DNA samples of BD and healthy controls were extracted by using the rapid genomic DNA extraction (RGDE) method from the peripheral blood collected in tubes containing EDTA. Total RNA was extracted from the PBMCs according to the protocol of TRIZOL® reagent (Invitrogen, San Diego, CA). The quality and concentration of RNA was measured by spectrophotometry method (Nano drop ND1000 at 260‐280 nm). The entirety of total RNA of the individual samples was shown by 1% agarose gel electrophoresis. Complementary DNA (cDNA) samples were synthesized using the reverse transcription reagent kit (Thermo Fisher Scientific) and real‐time polymerase chain reaction method according to manufacturer's guidelines.

#### 2.2.2 Primer design

**Figure 2:**
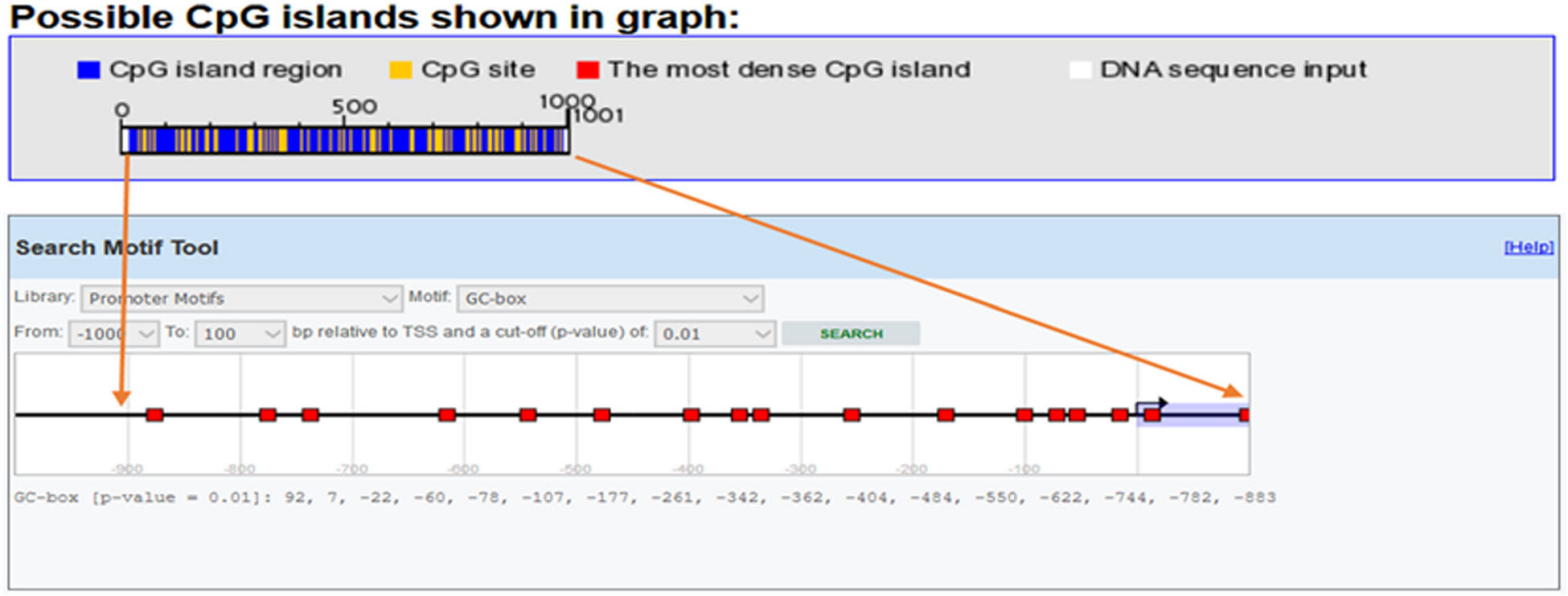
Diagram of VDR gene promoter loci in the DNA sequence (−800 to +200) for methylation: CpG site and island around TSS were predicted by EPD and CpG island Finder. In the diagram, CpG island region shown with blue, CpG site with yellow and the densest CpG Island with red colour. EPD, eukaryotic promoter database; TSS, transcription start site upstream; VDR, vitamin D receptor

A sequence of VDR promoter data was chosen from the National Center for Biotechnology Information (NCBI) and Ensemble (http://asia.ensembl.org/) databases. CpG islands of VDR promoter were predicted with eukaryotic promoter database (EPD). One pair of primers was designed using Primer Quest tool to amplify the CpG islands of transcription start site upstream (TSS) (Table 1; Figure 2).

**Table 1:** Primer design: A sequence of VDR promoter data was chosen from the Information NCBI and Ensemble databases. CpG islands of VDR promoter were predicted with EPD. One pair of primers was designed using Primer Quest tool

	Sequence of primer (5′‐3′)	Length	Tm	GC%
**VDR for gene expression**	**TCTCCTGCCTACTCACGATAA**	**21**	**54**	**47.6**
	**GCTACTGCCCGTGAGAATATAA**	**22**	**54**	**45.45**
**Primer for Β‐actin**	**GGTGAAGGTGACAGCAGT**	**18**	**55**	**50**
	**TGGGGTGGCTTTTAGGAT**	**18**	**55**	**45**
**VDR for MeDIP**	**CAGCGGTAAACTTGGCTACT**	**20**	**62**	**50**
	**CACGAACTTCAGCTTTCTCAAAC**	**23**	**62**	**43.5**

Abbreviations: EPD, eukaryotic promoter database; NCBI, National Center for Biotechnology Information; VDR, vitamin D receptor.

#### 2.2.3 RNA expression, real‐time polymerase chain reaction (Real‐Time PCR)

The following sequences of primers for the amplification of VDR were used: forward 5′‐TCTCCTGCCTACTCACGATAA‐3′ and reverse 5′‐GCTACTGCCCGTGAGAATATAA‐3′. Β‐actin was used as endogenous housekeeping gene and its expression was evaluated by the following primers: forward 5′‐GGTGAAGGTGACAGCAGT‐3′ and reverse 5′‐TGGGGTGGCTTTTAGGAT‐3′.

The Real‐time PCR Detection System was used for the amplification of cDNA through the working cycling platform as follows: denaturation was conducted at 94°C for 3 minutes followed by 42 cycles of denaturation for 10 seconds at 94°C; the annealing stage was performed for 35 seconds at 54°C; the extension stage was carried out for 20 seconds at 72°C followed by the final extension at 72°C for 10 minutes.

The pureness of PCR products was measured by dissociation curve plots. Amplification plots were used to assign values for the “cycle threshold” (Ct) by SLAN software. The analysis of the relative expression level of VDR was calculated by the 2^ΔΔCt^ method to produce the data as fold change up/down regulation.

#### 2.2.4 MeDIP‐QPCR technique and DNA promoter methylation

The extracted DNA is randomly sheared by sonication to generate fragments between 200 bp and 800 bp using the BANDELIN sonicate (UVV: 3200, Germany) for 15 cycles of 20 seconds on/20 seconds off. Fragment size was checked by electrophoresis on a 1.5% agarose gel and stained with ethidium bromide.

Methylated DNA immunoprecipitation (MeDIP) was carried out using EpiQuik™ MeDIP Ultra Kit (Epigentek, Farmingdale, NY, Cat No: P‐1052) according to manufacturer's guidelines. The sonicated DNA is immunoprecipitated with a monoclonal antibody against 5‐methylcytosine (5mC). Immunoprecipitation stage of 5mC enriched DNA fragments is processed at 4°C for 2 hours using 1 μL of monoclonal antibody against 5‐mC for the sample and 1 μL of normal mouse IgG as the negative control in a final volume of 100 mL antibody buffer. A portion of the sonicated DNA should be left untreated to serve as input control.

MeDIP reactions were set up in a 0.5‐mL vial by adding 84 μL of MeDIP buffer, 10 μL of sample DNA (ng/μL), 5 μL of BS (Blocker Solution), and 1 μL of 5‐mC Ab. The same steps were followed for the negative control using 1 μL of nonimmune IgG. The contents of the vials were incubated at room temperature for 60 minutes on a rolling shaker for 1 hour. Then the contents of the vials were transferred to the strip‐wells and incubated for 60 minutes at room temperature. Immune complexes were resuspended in proteinase K (60°C for 20 minutes) to release the methylated DNA fragments from the antibodies. The eluted DNA was stored at –20°C for later use in the real‐time qPCR.

### 2.3 Quantitative Real‐Time PCR reactions

Quantitative Real‐Time PCR of *VDR* gene was performed on the MIC real‐time instrument according to the manufacturer's recommendations. The following specific sequences of primers of VDR for MeDIP were used: forward 5′‐CAGCGGTAAACTTGGCTACT‐3′ and reverse 5′‐CACGAACTTCAGCTTTCTCAAAC‐3′.

To perform real‐time PCR reactions, 1 μL of eluted DNA, 1 μL from each of forward and reverse primer (0.5 μM), 10 μL of Master Mix, and 7 μL of DNA/RNA‐free water were used.

To promote the PCR amplification conditions, activation was conducted at 95°C for 2 minutes followed by denaturation at 95°C for 5 minutes; then 40 cycles of denaturation were carried out for 10 seconds at 95°C; then annealing was performed for 10 seconds at 55°C followed by extension for 10 seconds at 72°C; the final extension was performed at 72°C for 1 minute. The enrichment efficiency level of methylated DNA was determined as fold enrichment (FE) according to this formula:

$$\mathrm{FE}\;\%=2^{(\mathrm{IgG}\;\mathrm{CT}‐\;\mathrm{Sample}\;\mathrm{CT})\;x\;100}$$

### 2.4 Statistical analysis

A statistical analysis was performed using SPSS software version 16.0 (SPSS, Chicago, IL). The normal distributions of the data were tested with the Kolmogorov‐Smirnov test with Lilliefors correction. The quantitative data were presented as mean ± standard deviation (SD). Independent‐samples t test and Mann‐Whitney U test were used for assessing any difference in methylation pattern and messenger RNA expression levels of VDR between BD and control groups. A *P*‐value of less than .05 (*P* < .05) was accepted as a statistically significant difference.

## 3 RESULTS

Forty‐eight BD patients and 60 healthy controls participated in this study. The demographic and clinical characteristics of the participants are presented in (Table 2). The mean age (year's ± SD) and female/male ratio in BD and control groups are 38.1 ± 10.3, 19/29, and 37.48 ± 7.4, 23/37, *P* > .05 respectively. No significant difference was observed in age and gender between patients with BD and controls.

**Table 2:** The demographic and clinical characteristics of the participants

Clinical characteristics	Behcet's disease group (N = 48)	Healthy control group (N = 60)	*P*‐value
Age (mean ± SD) y	38.1 ± 10.3	37.4 ± 8.5	NS
Gender (male:female)	29/19 (1.5)	37/23 (1.6)	NS
Oral aphthous ulcer (%)	46 (95.8)	…	…
Uveitis (%)	28 (58.3)	…	…
Genital ulcer (%)	24 (50)	…	…
Positive pathergy (%)	19 (39.6)	…	…
Pseudo folliculitis (%)	11 (22.9)	…	…
Arthritis (%)	9 (18.8)	…	…
Erythema nodosum (%)	8 (16.7)	…	…
Phlebitis (%)	5 (10.4)	…	…
CNS involvement	1 (2.1)	…	…
HLA‐B5 (%)	27 (56.3)	…	…
HLA‐B51 (%)	25 (52.1)	…	…
Medications used		…	…
Colchicine (%)	25 (52.1)	…	…
Prednisolone (%)	23 (47.9)	…	…
Azathioprine (%)	22 (45.8)	…	…
Methotrexate (%)	15 (31.3)	…	…
NSAIDs (%)	12 (25)	…	…
Interferon‐α (%)	2 (4.2)	…	…
Cyclophosphamide (%)	2 (4.2)	…	…
Cyclosporine (%)	1 (2.1)	…	…
Sulfasalazine (%)	1 (2.1)	…	…

Abbreviations: CNS, central nerves system; HLA, human leukocyte antigen; NSAID, non steroid anti‐inflammation drugs; SD, standard deviation.

### 3.1 Real‐time quantitative PCR

**Figure 3:**
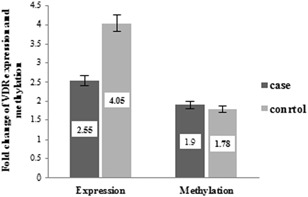
Fold changes of VDR expression levels and methylation pattern. As shown in the chart, the expression level of *VDR* gene was significantly lower in BD patient compared with the healthy group (*P* = .013). There was no significant difference between promoter methylation patterns of *VDR* gene in BD patient compared with the control group (*P* = .72). BD, Behcet's disease; VDR, vitamin D receptor

The results of the quantitative real‐time PCR analysis showed that *VDR* gene expression levels of the patients with BD were significantly lower than those in the control group (2.55 ± 2.71 and 4.04 ± 5.81, respectively, *P* = .013) (Figure 3).

Also, we analyzed the association between VDR gene expression and demographic and clinical features in the patient groups. As the results show, the expression level of *VDR* gene was significantly different between female and male in patient group (*P* = .001). *VDR* gene expression was also significantly increased in individuals with phlebitis (*P* = .04) (Table 3). There was no significant difference between expression level of *VDR* gene and other demographic and clinical features in the patient groups (*P* > .05).

**Table 3:** Clinical profile of *VDR* gene expression and methylation in patients with BD

Characteristics and clinical features		Frequency	Change fold of VDR expression (mean ± SD)	*P*‐ value	Change fold of VDR methylation (mean ± SD)	*P*‐value
Age	<45	25 (52.1)	2.94 ± 2.81	.301	1.79 ± 1.01	.556
	≥45	23 (47.9)	2.01 ± 1.85		1.90 ± 0.09	
Gender	Male	29 (60.4)	1.58 ± 1.46	**.001**	1.85 ± 0.57	.975
	Female	19 (39.6)	4.25 ± 2.70		1.74 ± 0.65	
HLA‐B5	Positive	27 (56.3)	2.66 ± 3.20	.716	1.78 ± 0.54	.292
	Negative	21 (43.7)	2.27 ± 2.65		2.05 ± 0.69	
HLA‐B51	Positive	25 (52.1)	2.66 ± 2.26	.877	1.72 ± 0.92	.213
	Negative	23 (47.9)	2.42 ± 2.03		2.16 ± 0.44	
Arthritis	Positive	9 (18.8)	1.27 ± 1.22	.167	1.57 ± 0.47	.167
	Negative	39 (81.2)	2.83 ± 2.91		1.98 ± 0.93	
Oral aphtha	Positive	26 (59.1)	0.143 ± 0.157	.729	1.082 ± 0.251	.962
	Negative	18 (40.9)	0.114 ± 0.09		1.230 ± 0.302	
Genital ulcer	Positive	24 (50)	2.96 ± 2.98	.317	2.01 ± 0.96	.180
	Negative	23 (50)	2.17 ± 2.45		1.80 ± 0.79	
Pathergy	Positive	19 (39.6)	2.56 ± 2.67	.506	1.77 ± 0.35	.876
	Negative	29 (60.4)	3.36 ± 3.26		1.84 ± 0.79	
Phlebitis	Positive	5 (10.4)	4.88 ± 3.72	**.04**	1.75 ± 0.64	.51
	Negative	43 (89.6)	2.33 ± 2.22		1.85 ± 0.57	
Severe BD	Positive	26 (55.3)	0.142 ± 0.141	.416	1.172 ± 0.134	.299
	Negative	21 (44.7%)	.103 ± 0.082		1.165 ± 0.40	
Uveitis	Positive	28 (58.3)	2.23 ± 1.88	.306	1.84 ± 0.64	.826
	Negative	20 (41.7)	3.14 ± 2.48		1.89 ± 0.69	
Vision loss	Positive (one eye)	5 (12.2)	0.141 ± 0.103	.656	1.054 ± 0.192	.320
	Negative	36 (87.8)	0.130 ± 0.127		1.180 ± 0.306	
Skin lesions	Positive	18 (37.5)	2.15 ± 0.78	.482	1.88 ± 1.67	.802
	Negative	30 (62.5)	3.40 ± 0.78		1.78 ± 0.52	

*Note*: As shown in the chart, the expression level of VDR gene was significantly different between female and male in patient group (*P* = .000). *VDR* gene expression was also significantly increased in individuals with positive phlebitis (*P* = .04). There was no significant difference between expression level of *VDR* gene and other demographic and clinical features in the patient group (*P* > .05). There was no significant difference between promoter methylation patterns of *VDR* gene in BD patient group (*P* > .05).

Abbreviations: BD, Behcet's disease; HLA, Human leukocyte antigen; SD, standard deviation; VDR, vitamin D receptor.

### 3.2 Evaluation of the DNA promoter methylation levels

**Figure 4:**
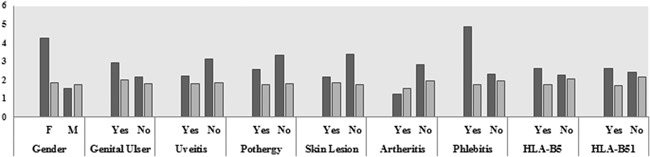
Evaluation of the association between the expression level and promoter methylation of the *VDR* gene with clinical features in the patient group. F, female; HLA, Human leukocyte antigen; M, male; SD, standard deviation; VDR, vitamin D receptor

Genomic DNA samples of the BD patients and healthy controls were evaluated by MeDIP‐QPCR technique. As our results show, there was no significant statistically difference between methylation pattern in the promoter regions of VDR in BD patient compared with the control group (1.90 ± 0.87 and 1.78 ± 0.63, respectively, *P* = .72) (Figure 3). Also, we analyzed the association between the promoter methylation pattern of the VDR and demographic and clinical features in the patient groups (Table 3). There was no significant difference between methylation patterns of VDR and other demographic and clinical features in the patient group (*P* > .05) (Figure 4).

As shown in the chart, the expression level of *VDR* gene was significantly different between female and male in the patient group (*P* = .000). *VDR* gene expression was also significantly increased in individuals with positive phlebitis (*P* = .04) There was no significant difference between expression level and methylation of *VDR* gene with other demographic and clinical features in the patient groups (*P* > .05).

No correlation was observed between *VDR* gene expression and BD activity, which was measured by BDCAF score (*r* = −0.054, *P* = .750), IBDDAM (*r* = −0.033, *P* = .834), and TIAI (*r* = −0.246, *P* = .104). In addition, the correlations between VDR DNA methylation and BDCAF (*r* = −0.127, *P* = .446), IBDDAM (*r* = 0.262, *P* = .086), and TIAI (*r* = 0.149, *P* = .329) were not significant.

## 4 DISCUSSION

Our study showed a decrease in VDR gene expression in the patients with BD, while DNA methylation pattern in the promoter regions of VDR was similar in the BD and control groups. We found a lower expression of VDR gene in males compared with females and a higher expression of *VDR* gene in individuals with phlebitis. No correlation was observed between *VDR* gene expression and DNA methylation with BD activity.

Expression of VDR is regulated by genetic, hormonal, epigenetic, and environmental factors. A lower expression of VDR has been reported in some polymorphisms of VDR like FokI.^41^ Selvaraj et al.^42^ reported lower levels of VDR expression in PBMCs of homozygotes for “B” allele of BsmI and “t” allele of TaqI. Regulation of *VDR* gene by hormones including 1,25(OH)2D3, parathyroid hormone, retinoic acid, and the glucocorticoids is known.^43^ 1, 25(OH)2D3 increases the level of *VDR* gene expression.^44^

Epigenetic mechanisms reversibly change the gene transcription without changing DNA sequences. The most known epigenetic mechanisms are DNA methylation and chromatin remodeling and histone acetylation.^14^ DNA methylation downregulates *VDR* gene expression, while histone methylation is able to repress as well as activate the VDR gene transcription.

Environmental factors (like diet, sun exposure, pollutions, and infections) regulate the VDR by altering the vitamin D levels. 1α,25(OH)2D3 after binding to VDR regulates VDR expression by histone modification and DNA methylation or demethylation.^45^ Many studies showed an association between *VDR* gene, DNA hypermethylation, and malignancies and infectious diseases.^46^, ^47^ Marik et al^48^ reported hypermethylation of VDR promoter and low VDR in breast cancer cells. They showed that methylation of exon1a of the VDR is significantly higher (65% of CpGs is methylated) than normal breast tissue (15% of CpGs is methylated).^48^ In contrast, in vitro study in colon cancer cell lines did not show any promoter region methylation of the VDR, and treatment with AZA did not increase gene expression.^14^ Similarly, decreased VDR expression without any alterations in methylation was reported in parathyroid tumors.^23^, ^49^

The most remarkably VDR‐related infectious diseases include human immunodeficiency virus (HIV), tuberculosis (TB), and leprosy.^47^ In vitro studies by Chandel et al,^50^ showed that the infection of the previously activated T cells with HIV led to upregulation of DNA methyltransferases 3b (DNMT3B), increased promoter methylation of VDR (45%‐70%), and decreased *VDR* gene expression. They showed that treatment with 5‐azacytidine (AZA) could reverse downregulation of VDR. It means that the DNA methylation have some role in the low expression of VDR by HIV. The importance of VDR expression in the development of other diseases is becoming increasingly apparent. Kempinska‐Podhorodecka et al^20^ showed that expression of the VDR gene in PBMCs of patients with primary biliary cirrhosis and primary sclerosing cholangitis is decreased.^20^

Regarding the low expression of VDR gene in patients with BD, we could not find any significant association between methylation pattern and the expression level of *VDR* gene. More and larger studies on the molecular level are needed to determine the lower expression of *VDR* gene in patients with BD. The major limitation of our study was that we analyzed only the methylation of VDR DNA. Other epigenetic mechanisms may have been involved in the lower expression of VDR in the patients with BD.

## 5 CONCLUSION

*VDR* gene expression decreased in patients with BD. However, there is no suggestion evidence that the expression level of VDR is regulated by a unique DNA methylation mechanism. No correlation exists between *VDR* gene expression and BD activity.

## CONFLICT OF INTERESTS

The authors declare that there are no conflict of interests.
